# Supplementary material for: Latent profile analysis patterns of exercise, sitting and fitness in adults – Associations with metabolic risk factors, perceived health, and perceived symptoms
Source: PLoS One. 2020 Apr 24;15(4):e0232210. doi: 10.1371/journal.pone.0232210 (PMC7182226; doi:10.1371/journal.pone.0232210)
Supplement: S1 Table — (PDF) [file pone.0232210.s001.pdf]

S1 Table. Fit indices of the estimated latent profile analysis

Model	LL	#fp	CAIC	BIC	ABIC	Entropy
1 profile	-368753.746	8	737604.145	737596.145	737570.721	na
2 profile	-361914.799	13	723986.660	723973.660	723932.345	.693
3 profile	-358465.181	18	717147.832	717129.832	717072.628	.941
4 profile	-354996.390	26	710306.904	710280.904	710198.276	.685
5 profile	-354198.838	28	708735.963	708707.963	708618.978	.721
6 profile	-353225.896	33	706850.488	706817.488	706712.613	.711
7 profile	Converged on local solution (i.e., did not replicate the best log likelihood value)					

Note. LL = log-likelihood, #fp = number for free parameters, CAIC = consistent Akaike Information Criterion, BIC = Bayesian Information Criterion, and ABIC = sample-size adjusted BIC.
