# Supplementary material for: Latent profile analysis patterns of exercise, sitting and fitness in adults – Associations with metabolic risk factors, perceived health, and perceived symptoms
Source: PLoS One. 2020 Apr 24;15(4):e0232210. doi: 10.1371/journal.pone.0232210 (PMC7182226; doi:10.1371/journal.pone.0232210)
Supplement: S2 Table — (PDF) [file pone.0232210.s002.pdf]

S2 Table. Drop-out analysis comparing those excluded (n=19.892) and excluded (n=45.078) in multi-adjusted analyses in Fig 2.

	Excluded	Included	Difference
Sex (women)	42.1% (8377)	41.5% (18718)	p=0.161
Age (year)	42.9 (10.9)	43.4 (11.4)	p<0.0001
Profile			
1	11% (2144)	16% (7336)	
2	19% (3846)	16% (7381)	
3	17% (3425)	24% (10757)	
4	41% (8183)	33% (14834)	
5	9% (1760)	7% (3111)	
6	3% (534)	4% (1659)	p<0.0001
BMI (kg/m^2)	25.8 (4.2)	26.1 (4.3)	p<0.0001
Systolic BP (mmHg)	125.1 (14.8)	125.4 (15.0)	p=0.010
Diastolic BP (mmHg)	78.3 (10.4)	78.2 (10.5)	p=0.435
Healthy diet	69.8% (13873)	70.0% (31540)	p=0.657
Non-smokers	85.8% (17049)	84.7% (38198)	p<0.0001
Back/Neck pain (often/very often)	19.0% (3777)	19.9% (8954)	p=0.010
Overall stress (often/very often)	13.8% (2749)	12.6% (5685)	p<0.0001
Global health (poor/very poor)	5.9 % (1165)	5.9% (2645)	p=0.956
Sleeping problems (often/very often)	8.9% (987)	9.5% (3107)	p=0.046

Data is presented as mean (SD) or % (n). Differences between continuous variables and percentages were tested using independent t-tests and chi^2 test, respectively.
