# Supplementary material for: Latent profile analysis patterns of exercise, sitting and fitness in adults – Associations with metabolic risk factors, perceived health, and perceived symptoms
Source: PLoS One. 2020 Apr 24;15(4):e0232210. doi: 10.1371/journal.pone.0232210 (PMC7182226; doi:10.1371/journal.pone.0232210)
Supplement: Attachment — Submitted filename: PONE-D-19-27066_Reviewer_Comments.docx [file pone.0232210.s003.docx]

The purpose of this manuscript was to identify and describe the characteristics of naturally occurring patterns of sedentary time at work and in leisure time, exercise and cardiorespiratory fitness, and the association of such profiles with metabolic risk factors, perceived health and perceived symptoms.

**Point 1: Title and Short Title:** I suggest that the authors change the titles, replacing the word "physical activity" with "exercise". For in this study the authors evaluate the pattern of exercise and not of physical activity. Although such terms are still commonly used as synonyms, they have different meanings. Every exercise is a physical activity, but not every physical activity is an exercise.

**Point 2: Conclusions: Lines 47-50:** I think that the conclusion can be improved. The conclusion always has to focus on answering the study questions.

**Point 3: Introduction: Lines 54-55:** The authors report that “… sustained or decreased levels of exercise [6] …”. This reference refers to the study elaborated by Alan G. Knuth and Pedro C. Hallal entitled: Temporal Trends in Physical Activity: A Systematic Review. This study reported the trend regarding physical activity and not about exercise. Moreover, it seems to me that in the introduction and throughout the manuscript the authors used the terms "physical activity" and "exercise" as synonyms. I suggest that only the term "exercise" be used, as it was this domain that was evaluated in this study. It may be interesting to explain in the introduction the difference between the definitions of these terms: "physical activity" and "exercise".

**Point 4: Introduction: Lines 56-57:** Objective studies have identified that exercise represents a small portion of daily physical activity. Therefore, I think the following statement should be changed. “… resulting in greater variability in daily physical activity patterns and physical performance between individuals.”.

**Point 5: Material and methods:** I think the authors should inform in the session "Material and methods" the experimental design used in this study.

**Point 6: Material and methods: Lines 92-93:** The authors report that the participants answered an extensive questionnaire about physical activity habits. How reliable and reproducible is this questionnaire to identify daily physical activity and exercise?

**Point 7: Other variables: Lines 151-57:** The authors report that “… Physical activity level prior to the age of 20 years was self-reported by selecting …”. This variable has no reference. It would be interesting for the authors to indicate the reference for the elaboration of this variable. In this variable, is only considered the physical education classes held in the school environment? Another question I have is about the power of the physical education class to represent the level of physical activity prior to 20 years.

**Point 8: Results: Line 222:** What does “cf.” mean?

**Point 9: Results: Lines 285-86:** The authors report that “Profile 4 had in general a more beneficial health and symptom profile than profile 3, as did profile 6 compared to profile 5”. In table 2, the numbers show that profile 5 has lower results than profile 6. Therefore, from table 2, profile 5 is better (more beneficial) than 6. Please review this.

**Point 10: Discussion: Line 336:** The authors report: “physical activity patterns”. Although every exercise is a physical activity, not every physical activity is an exercise. What was subjectively assessed was exercise. So, I think it is more appropriate to use "exercise pattern".

**Point 11: Discussion: Lines 334-45:** The authors report: “Rather than examining interactions between different types of domains or proxies of physical activity, as we do in the present study …”. I could not find in this paper the analysis of the interactions between the different types of domains or proxies of physical activity, since the present study only assessed exercise.

**Point 12: Discussion: Lines 350-52:** The authors report: “Accumulating the recommended amount of weekly physical activity was consistently associated with a more beneficial metabolic health profile.”. Does this statement refer to data from the present study or citation data # 14 or # 29?

**Point 13: Discussion: Lines 352-53:** The authors report: “The manner in which this physical activity was accumulated (spread over the week or in just a few days) was largely associated with metabolic risk [29].” I suggest revising this sentence, as the reference cited reports the opposite: *“However, the manner in which this activity is accumulated, either spread over most days of the week or compressed into just a couple of days, may have similar associations with the risk factors for the MS. (Am J Health Promot 2010;24[3]:161–169.)”*.

**Point 14: Discussion: Line 354:** The authors report “where time spent in more physical activity”. I think the authors should state what "more physical activity" means and what intensity of physical activity, because such information is very important.

**Point 15: Discussion: Lines 355-56:** The authors report: “regardless of how physical activity time was accumulated over the week [12].”. This sentence contradicts what is written in the sentence of lines 352-53.

**Point 16: Discussion: Lines 358 and 360:** I suggest changing the term "physical activity" to "exercise".

**Point 17: Discussion: Lines 359-60:** The authors report: “… we show a general dose-response in the association between the profiles accumulating more physical activity and less sedentary time …”. I could not find in the text what would be the minimum necessary physical activity, exercise and sedentary time that should be adopted. In addition, I reinforce that the study evaluated exercise and not general physical activity. When considering dose response, we should take into account that the recommendations of physical activity, exercise and more recently sedentary behavior (at least for children and adolescents), the minimum amount of such behaviors for different age groups (children, adolescents, adults and the elderly) vary considerably. Thus, as the present study is composed of adults and the elderly, it might be more interesting to separate these groups.

**Point 18: Discussion: Lines 369-70:** As the authors previously reported: Hansen et al. [reference #15] identified and characterized patterns of physical activity. I think we should be very careful to say that: “Comparing these profiles reveal similar characteristic patterns for …”. Since the present study evaluated exercise and not total physical activity. I suggest reviewing the final part of this paragraph.

**Point 19: Discussion: Lines 392-93:** The authors report: “… we identify patterns of naturally occurring interactions of four different domains of physical activity …”. I could not identify in the manuscript the four different domains of physical activity.

**Point 20: Discussion: Lines 403-406:** Although the authors have presented such limitations for this study, I believe there was a lack of warning about how these limitations may impact the interpretation of the results presented.

**Point 21: Discussion:** I would like to suggest that the authors address more the clinical relevance of these findings within the discussion.

**Point 22: Conclusions: Line 413:** I suggest changing the term "physical activity" to "exercise".

**Point 23: Conclusions: Lines 414-15:** The authors report: “and not only overall physical activity level …”. I disagree with the author's statement. For the public health message that must be propagated is that of stimulating the increase of physical activity levels, regardless of the type and intensity. I suggest reading the following papers:

Klenk J, Kerse N. Every step you take. BMJ. 2019;366:l5051. doi: 10.1136/bmj.l5051.

Ekelund U, Tarp J, Steene-Johannessen J, Hansen BH, Jefferis B, Fagerland MW, et al. Dose-response associations between accelerometry measured physical activity and sedentary time and all cause mortality: systematic review and harmonised meta-analysis. BMJ. 2019;366:l4570. doi: 10.1136/bmj.l4570.

**Point 24: Conclusions:** I suggest improving the wording of the conclusion and trying to respond to the objectives.
